# mRNA vaccine against SARS-CoV-2 response is comparable between patients on dialysis and healthy controls after adjustment for age, gender and history of COVID-19 infection

**DOI:** 10.1007/s40620-024-02161-w

**Published:** 2024-12-20

**Authors:** Guy Rostoker, Stéphanie Rouanet, Myriam Merzoug, Hiba Chakaroun, Mireille Griuncelli, Christelle Loridon, Ghada Boulahia, Luc Gagnon

**Affiliations:** 1 Department of Nephrology and Dialysis, Ramsay Santé, Hôpital Privé Claude Galien, Quincy-sous-Sénart, France; 2 Collège de Médecine des Hôpitaux de Paris, Paris, France; 3 StatEthic, Levallois-Perret, France; 4 Infection Prevention and Control Registered Nurse, Ramsay Santé, Hôpital Privé Claude Galien, Quincy-sous-Sénart, France; 5 IQVIA Laboratories Vaccines, Laval, Canada

**Keywords:** Anti-spike antibodies, Dialysis, Healthy controls, Neutralizing antibodies, SARS-CoV-2

The recent coronavirus disease 2019 pandemic (COVID-19) triggered an unprecedented health crisis in the world. In particular, it had a major impact on the management of dialysis patients, in whom high mortality was seen in the first wave of the pandemic [1]. Mass vaccination against SARS-CoV-2, especially with messenger RNA (mRNA) vaccines reduced hospitalization and mortality both in the general and in the end-stage kidney disease (ESKD) populations [2, 3]. Age and a history of previous COVID-19 disease appear to be major factors of vaccine response (both in the general population and in ESKD patients) [1–3] which is best appreciated in the month(s) following the second dose (or a single dose in those who had previous COVID-19 infection). Neutralizing antibodies and anti-spike antibodies are seen by the Federal Drug Administration (FDA) in the US and the European Medicines Agency (EMA) as surrogate markers and valuable correlates of protection [2, 4]. Immunological response (evaluated by anti-spike antibody assays) to full vaccination has been repeatedly shown to be robust in ESKD patients on dialysis (unlike in renal transplant patients) but of lesser magnitude (and earlier waning) than in healthy controls (who were generally far younger) [5, 6]. We hypothesize that age, gender, and a history of COVID-19 could be factors strongly influencing this difference and we suspect that statistical adjustments may strongly affect this difference.

This retrospective serological study aimed to analyze the quality and determinants of vaccine response evaluated within 1–3 months following a full vaccination with 2 doses of the Pfizer original mRNA-based COVID-19 vaccine (COMIRNATY). It was conducted in 2021 (as soon as vaccines became available in France), in a cohort of patients treated in the dialysis center of Claude Galien Hospital and in healthy controls belonging to the medical and nursing staff of the dialysis unit.

In accordance with French law, approval by an ethics committee was not required for this retrospective observational study. This study falls within the framework of the MR-004 reference methodology defined by the Commission Nationale de l’Informatique et des Libertés (CNIL), France’s national authority for protecting data privacy. The study was registered on the public health data hub (registration number N° F20220207122956) after being approved by the scientific college of the Ramsay Health Cooperation Group for Education and Research (GCS Ramsay Santé), which was responsible for this study (COS-RGDS-2021-06-075). All participants gave informed oral consent. All data were analyzed unless patients refused to participate in the study.

Levels of anti-SARS-CoV-2 spike antibodies were measured during the routine biological follow-up of patients by the SELAS Cerballiance laboratory (Ile-de-France Sud, Lisses, France) using Chemiluminescence ALINITY from Abbott, and were initially expressed in UA/mL (later transposed for this study into BAU/mL using the equation BAU/mL = 1/7 UA/mL). Neutralizing antibodies (NA) against SARS-CoV-2 (the ancestral Wuhan virus and its Delta and Omicron BA1 variants) were measured by NEXELIS (Laval, Canada) using pseudo-typed virus neutralization assays as previously described [7] on – 90° Celsius frozen twin serum samples stored as required by French health regulations. Values of neutralizing antibodies were expressed in UA (inverse dilution able to inhibit 50% of cell cultures infected with pseudo-viruses). Protection threshold for neutralizing antibodies was set at ≥ 32 UA as previously described in Knell et al. [8].

Continuous variables were reported as median values and interquartile ranges [IQR]. Categorical variables were reported as numbers and percentages. Anti-SARS-CoV-2 spike antibodies and neutralizing antibodies against SARS-CoV-2 were compared between the ESKD patients treated by dialysis and the healthy controls, firstly by using the Wilcoxon–Mann–Whitney test on raw data, and secondly, by using the Wilcoxon–Mann–Whitney test on data (in log) adjusted for age, sex and previous COVID-19 infection, i.e. after removing the effect of age, sex and previous COVID-19 infection (in case of a sufficient number of patients or controls) on the anti-SARS-CoV-2 spike antibodies and neutralizing antibodies against SARS-CoV-2 [9]. Correlations between anti-SARS-CoV-2 spike antibodies and neutralizing antibodies against SARS-CoV-2 were assessed using the Spearman’s rank correlation coefficient with 95% two-sided confidence interval (CI) [9]. The proportion of ESKD patients treated by dialysis with neutralizing antibodies above or equal to the protection threshold was described with 95% two-sided CI (Wilson method). Correlation coefficients were interpreted as weak (0.1 ≤ |rho| < 0.3), moderate (0.3 ≤ |rho| < 0.5) or strong (|rho|≥ 0.5) [9]. For all statistical tests, a *p* value < 0.05 was considered to denote statistical significance. All statistical analyses were performed using R Statistical Software version 4.3.1.

This study included 125 ESKD patients treated by dialysis (hemodialysis *n* = 123; peritoneal dialysis *n* = 2; 60.8% men; median age: 68 [56–78]; previous COVID-19 infection: 34.4%; median Charlson comorbidity index: 7 [5–8]) and 17 healthy controls (11.8% men; median age: 34 [28–39]; previous COVID-19 infection: 47.1%). Their main characteristics are shown in Supplementary Tables 1 and 2, and include the patients’ main comorbidities and causes of kidney failure (Supplementary Table 1).

Median anti-spike IgG after a second dose of vaccine was 746 BAU/ml [150–3395] in ESKD patients as compared to 1531 BAU/ml [1016–3619] in healthy controls (*p* = 0.054) (Fig. 1a). Interestingly, after adjustment, the residual part of anti-spike IgG not explained by age, gender and previous COVID-19 infection did not significantly differ between patients on dialysis and healthy controls (*p* = 0.78) (Fig. 1b). Moreover, the titer of neutralizing antibodies against the ancestral Wuhan viral strain and its Delta and Omicron BA1 variants did not differ statistically, either on raw data or after adjustment (*p* > 0.05) (Wuhan neutralizing antibodies: controls (*n* = 7): 278 [214–872] UA; patients (*n* = 44): 268 [153–842] UA, Fig. 1c and d; Delta neutralizing antibodies: controls (*n* = 10): 99 [95–807] UA; patients (*n* = 90): 48 [14–739] UA, Fig. 1e and f; Omicron BA1 neutralizing antibodies: controls (*n* = 7): 20 [9–46] UA, patients (*n* = 43): 23 [0–92] UA, Fig. 1g and h).

**Fig. 1:**
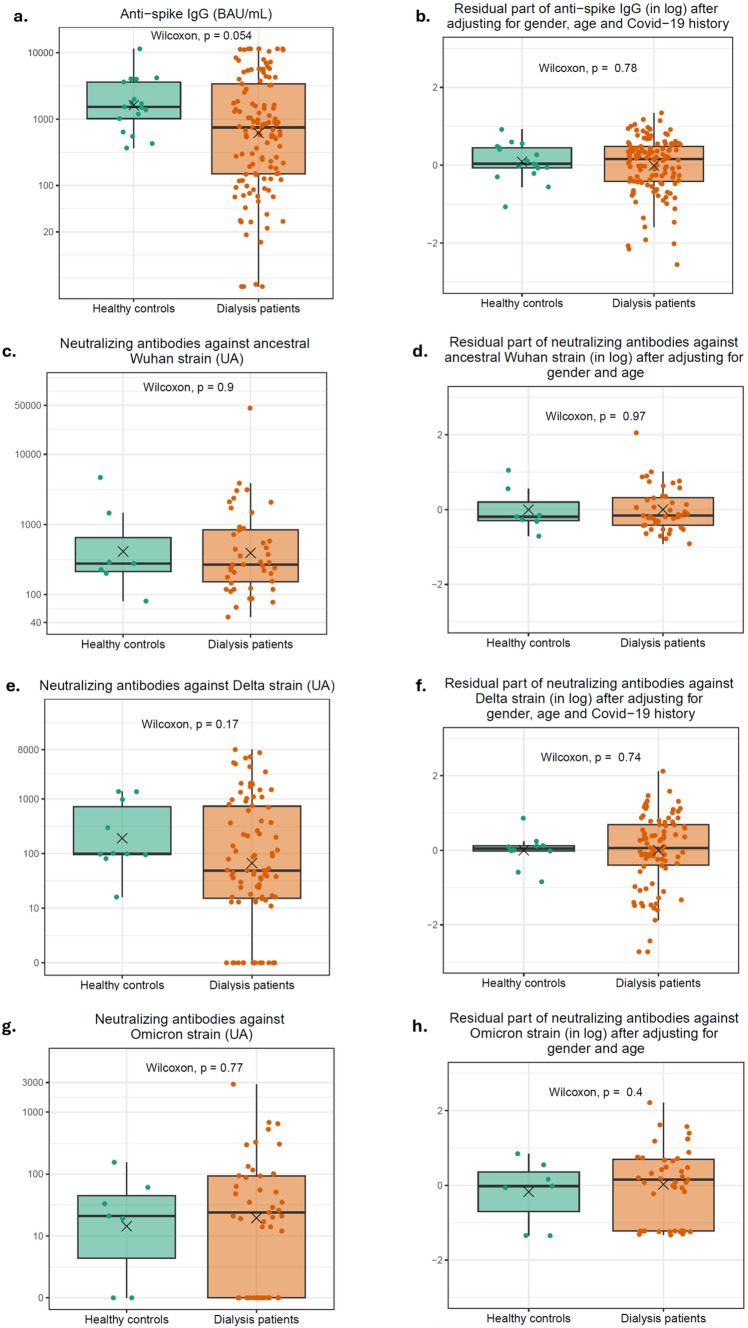
Comparison of antibodies in controls and dialysis patients after the second dose. **a** Anti-spike antibodies in controls (*n* = 17) and dialysis patients (*n* = 125) raw data. **b** Anti-spike antibodies in controls (*n* = 17) and dialysis patients (*n* = 125) after adjustment (residuals are derived from the following linear regression $\text{log}\left(IgG\right)={\alpha }_{0}+{\alpha }_{1}*\text{gender}+{\alpha }_{2}*age+{\alpha }_{3}*{\text{covid}}_{19}\text{ history}+\text{residual}$). **c** Neutralizing antibodies against ancestral Wuhan strain in controls (*n* = 7) and dialysis patients (*n* = 44) raw data. **d** Neutralizing antibodies against ancestral Wuhan strain in controls (*n* = 7) and dialysis patients (*n* = 44) after adjustment (residuals are derived from the following linear regression $\text{log}\left(\text{Wuhan}\right)={\alpha }_{0}+{\alpha }_{1}*\text{gender}+{\alpha }_{2}*age+\text{residual}$). **e** Neutralizing antibodies against Delta strain in controls (*n* = 10) and dialysis patients (*n* = 90) raw data. **f** Neutralizing antibodies against Delta strain in controls (*n* = 10) and dialysis patients (*n* = 90) after adjustment (residuals are derived from the following linear regression $\text{lo}g\left(Delta\right)={\alpha }_{0}+{\alpha }_{1}*\text{gender}+{\alpha }_{2}*age+{\alpha }_{3}*{\text{covid}}_{19}\text{ history}+\text{residual}$). **g** Neutralizing antibodies against Omicron strain in controls (*n* = 7) and dialysis patients (*n* = 43) raw data. **h** Neutralizing antibodies against Omicron strain in controls (*n* = 7) and dialysis patients (*n* = 43) after adjustment (residuals are derived from the following linear regression $\text{log}\left(\text{Omicron}\right)={\alpha }_{0}+{\alpha }_{1}*\text{gender}+{\alpha }_{2}*\text{age}+\text{residual}$)

Of note, since blood samples for SARS-CoV-2 antibody measurement were collected in healthy controls after a median delay of 63 days [49–77], while blood samples in dialysis patients were collected for the same purposes after a median delay of 33 days [31–38], we evaluated the influence of the delay of sampling on antibody levels: additionally adjusting for the delay between vaccination and antibody quantification led to the same conclusion for each analysis (IgG anti-Spike *p* = 0.61; neutralizing antibodies against Wuhan strain *p* = 0.53; neutralizing antibodies against Delta strain *p* = 0.26; neutralizing antibodies against Omicron strain *p* = 0.35).

The proportion of patients with neutralizing antibodies above or equal to the protection threshold (32 UA) was 100% (95% CI [92–100]) for the Wuhan strain, 62.2% (95% CI [51.9–71.5]) for Delta and 44.2% (95% CI [30.4–58.9]) for Omicron BA1 variants. Finally, anti-Spike antibodies were highly correlated with neutralizing antibodies for the Wuhan ancestral strain (rho = 0.799; 95% CI [0.658–0.886]), the Delta variant (rho = 0.908; 95%CI [0.863–0.939]) and the Omicron BA1 (rho = 0.688; 95% CI [0.488–0.819]). Since the comorbidity profile of healthy controls was not available, it might have affected their antibody response to vaccine and weakened the conclusion of the study.

In conclusion, our results strongly suggest that the initial vaccine response to COVID-19 mRNA vaccines in patients on dialysis assessed by IgG anti-Spike antibodies and neutralizing antibodies is of the same magnitude as in healthy controls if demographic factors (age and gender) and history of COVID-19 infection are considered, although the relatively small size of our sample may limit generalization. This is consistent with the major impact of age on levels of neutralizing antibodies against SARS-CoV-2 in the general population. We would suggest two main recommendations of this study. First, further studies are warranted in larger population samples to confirm our findings. Second, additional studies could be conducted in the future to assess antibody response to different SARS-CoV-2 virus strains in people on dialysis.

## Supplementary Information

Below is the link to the electronic supplementary material.

Supplementary file1 (DOCX 21 KB)

Supplementary file2 (DOCX 14 KB)

Supplementary file3 (DOCX 33 KB)

## Data Availability

Deidentified and anonymized data will be made available upon reasonable request. Requests should be directed to rostotom@orange.fr.
