# Supplementary material for: mRNA vaccine against SARS-CoV-2 response is comparable between patients on dialysis and healthy controls after adjustment for age, gender and history of COVID-19 infection
Source: J Nephrol. 2024 Dec 20;38(1):301–4. doi: 10.1007/s40620-024-02161-w (PMC11903638; doi:10.1007/s40620-024-02161-w)
Supplement: Supplementary file 1 — Supplementary file1 (DOCX 21 KB) [file 40620_2024_2161_MOESM1_ESM.docx]

**Supplementary Table 1: Characteristics of 125 dialysis patients**

**Table 1a. Demographic and clinical characteristics**

|  | **Total (N=125)** |  | **Total (N=125)** | |
| --- | --- | --- | --- | --- |
| **Age (years)** |  | **Original kidney failure** |  | |
| Median | 68.0 | Hypertensive nephropathy | 32 (25.6%) |  |
| Q1 - Q3 | 56.0 - 78.0 | Diabetic nephropathy | 36 (28.8%) |  |
| **Sex** |  | Malformative uropathy | 14 (11.2%) |  |
| Female | 49 (39.2%) | Genetic kidney disease | 13 (10.4%) |  |
| Male | 76 (60.8%) | Immunological or general kidney disease | 19 (15.2%) |  |
|  |  | Undetermined kidney disease | 11 (8.8%) |  |
| **Weight (kg)** |  | **Diabetes** | 53 (42.4%) | |
| Median | 73.5 |  |  | |
| Q1 - Q3 | 65.5 - 85.0 |  |  | |
| **BMI (kg/m^2^)** |  | **Time spent on dialysis (months)** |  | |
| Median | 26.0 | Median | 35.7 | |
| Q1 - Q3 | 22.0 - 30.0 | Q1 – Q3 | 12.9 - 69.3 | |

BMI: Body Mass Index

**Table 1b. Dialysis characteristics**

|  | **Total (N=125)** |  | **Total (N=125)** |
| --- | --- | --- | --- |
| **Method of dialysis** |  | **Dialyse membrane** |  |
| HD (hemodialysis) | 69 (55.6%) | Polysulfone | 67 (54.9%) |
| HDF (hemodiafiltration) | 32 (25.8%) | PMMA (polymethyl methacrylate) | 19 (15.6%) |
| HDx (expanded hemodialysis) | 21 (16.9%) | AN69 (polyacrylonitrile) | 15 (12.3%) |
| PD (peritoneal dialysis) | 2 (1.6%) | MCO (medium cut-off) | 21 (17.2%) |
| Missing values | 1 | Missing values | 1 |
| **Dialyse bath** |  | **Vascular access** |  |
| AX (acetate) | 100 (82.0%) | Arteriovenous fistula | 84 (68.3%) |
| CX (citrate) | 22 (18.0%) | Catheter | 35 (28.5%) |
| Missing values | 1 | PTFE arteriovenous graft | 4 (3.3%) |
|  |  | Missing values | 0 |

PTFE: polytetrafluoroethylene

**Table 1c. Previous medical history**

|  | **Total (N=125)** |  | **Total (N=125)** |
| --- | --- | --- | --- |
| **Charlson comorbifity index** |  | **Charlson comorbidity index in class** |  |
| Median | 7.0 | 0 ≤ index < 4 | 12 (9.6%) |
| Q1 – Q3 | 5.0 - 8.0 | 4 ≤ index < 6 | 26 (20.8%) |
|  |  | 6 ≤ index < 8 | 47 (37.6%) |
|  |  | ≥8 | 40 (32.0%) |
| **Previous renal transplantation** | 13 (10.4%) | **Previous transplantation of other organs** | 0 (0%) |
| **Previous COVID infection prior to vaccination** | 43 (34.4%) | **Infection with COVID after the vaccination** | 17 (13.6%) |
| **Previous viral hepatitis B** |  | **Viral Hepatitis vaccine response** |  |
| Yes | 26 (20.8%) | Responder | 53/99 (53.5%) |
| No | 99 (79.2%) | Non-responder | 46/99 (46.5%) |

**Table 1d. Treatment of anemia**

|  | **Total (N=125)** | |  | | | **Total (N=125)** | | |
| --- | --- | --- | --- | --- | --- | --- | --- | --- |
| **Darbepoetin received in the year**  **prior to vaccination** | | 118/121 (97.5%) | | | **Cumulative darbepoetin dose (µg/week)** | | |  |
|  | |  | | Median | | | 38.5 |  |
|  | |  | | Q1 – Q3 | | | 25.0 - 67.0 |  |
|  | |  | | Missing values | | | 0 |  |
| **IV iron received in the year**  **prior to vaccination** | | 116 (95.9%) | | **IV iron dose (mg/month)** | | |  |  |
|  | |  | | Median | | | 267.0 |  |
|  | |  | | Q1 – Q3 | | | 171.0 - 346.0 |  |
|  | |  | | Missing values | | | 0 |  |
| **Transfusion received in the year**  **prior to vaccination** | | 29 (23.2%) | | **Number of RBC transfused in the year**  **prior to vaccination** | | |  |  |
|  | |  | | Median | | | 2.0 |  |
|  | |  | | Q1 – Q3 | | | 2.0 - 5.0 |  |
|  | |  | | Missing values | | | 0 |  |

IV: intravenous, RBC: red blood cell packs
