# Supplementary material for: mRNA vaccine against SARS-CoV-2 response is comparable between patients on dialysis and healthy controls after adjustment for age, gender and history of COVID-19 infection
Source: J Nephrol. 2024 Dec 20;38(1):301–4. doi: 10.1007/s40620-024-02161-w (PMC11903638; doi:10.1007/s40620-024-02161-w)
Supplement: Supplementary file 2 — Supplementary file2 (DOCX 14 KB) [file 40620_2024_2161_MOESM2_ESM.docx]

**Supplementary Table 2: Characteristics of 17 healthy controls**

|  | **Total (N=17)** |
| --- | --- |
| **Age (years)** |  |
| Median | 34.0 |
| Q1 - Q3 | 28.0 - 39.0 |
|  | |
| **Sex** |  |
| Female | 15 (88.2%) |
| Male | 2 (11.8%) |
|  |  |
| **Previous COVID infection prior to vaccination** |  |
| Yes | 8 (47.1%) |
| No | 9 (52.9%) |
|  | |

*Although the controls belong to the dialysis medical and paramedical staff, their eventual

comorbidities have not been studied (out of the scope of the study) and therefore, their Charlson

comorbidity index could not be calculated.
